# Bile Salt-Stimulated Lipase Activity in Donor Breast Milk Influenced by Pasteurization Techniques

**DOI:** 10.3389/fnut.2020.552362

**Published:** 2020-11-12

**Authors:** Jeewon Koh, Ashley F. Victor, Matthew L. Howell, Jooyoung G. Yeo, Yunyao Qu, Brandon Selover, Joy Waite-Cusic, David C. Dallas

**Affiliations:** ^1^Nutrition Program, School of Biological and Population Health Sciences, College of Public Health and Human Sciences, Oregon State University, Corvallis, OR, United States; ^2^Department of Food Science and Technology, College of Agricultural Sciences, Oregon State University, Corvallis, OR, United States

**Keywords:** bile salt-stimulated lipase, bile salt-activated lipase, donor breast milk, high-pressure processing (HPP), ultraviolet-C irradiation (UV-C), preterm infant

## Abstract

Breast milk contains bile salt-stimulated lipase (BSSL), which significantly increases the fat digestion capacity of newborns who have limited pancreatic lipase secretion in the first few months after birth. Problematically, Holder pasteurization used in non-profit milk banks to ensure the microbiological safety of donor milk for infants, particularly preterm infants (<37 weeks gestation age), destroys milk BSSL, thus limiting infant fat absorption capacity. Alternative strategies are needed to ensure the safety of donor milk while preserving BSSL activity. Three alternative pasteurization techniques—high-pressure processing (HPP, 550 MPa, 5 min), gamma cell irradiation (IR, 2.5 Mrads) and UV-C (254 nm, 0–33,000 J/L)—were compared with Holder pasteurization (low-temperature long-time, LTLT, 62.5°C, 30 min) for retention of BSSL activity in donor breast milk. As the time required for donor milk pasteurization by UV-C in published methods was not clear, donor breast milk was spiked with seven common bacterial strains and treated by UV-C for variable time periods and the minimum UV-C dosage required to achieve a 5-log_10_ reduction of CFU/mL was determined. Eight thousand two hundred fifty J/L of UV-C exposure was sufficient to achieve 5-log_10_ reduction of each of bacterial targets, including *Bacillus* and *Paenibacillus* spores. The retention of BSSL activity was highest after HPP (retaining 62% of the untreated milk BSSL activity), followed by UV-C (16,500 J/L), IR and LTLT (35, 29, and 0.3% retention, respectively). HPP was an effective alternative to pasteurize milk with improved retention of BSSL activity compared to Holder pasteurization. Future work should investigate the effect of alternative pasteurization techniques on the entire array of bioactive components in donor breast milk and how these changes affect preterm infant health outcomes. Implementation of HPP technique at milk banks could improve donor milk-fed infant fat absorption and growth.

## Introduction

Newborn infants have low fat digestion capacity for the first few months of life due to immature pancreatic development and low lipase secretion (1). Both term and preterm infants have low pancreatic lipase activity from birth to the first month of life compared with older children (2, 3). For preterm infants, 20–30% of human milk-derived dietary fat is not digested and absorbed in the first months of life (4). Impaired fat digestion can limit weight gain and infant development (4).

Breast milk contains bile salt-stimulated lipase (BSSL) that is activated by bile salts when milk reaches the infant's intestine and contributes to fat digestion ability for infants (4). Premature infants are often fed donor breast milk because their mothers often cannot produce adequate amounts of breast milk. Non-profit milk banks pasteurize [heating at 62.5°C for 30 min, also known as low-temperature long-time pasteurization (LTLT)] donor milk to inactivate bacteria and viruses. However, BSSL is completely degraded by Holder pasteurization (5–7). The degradation of BSSL by pasteurization further reduces overall fat absorption from human milk 17–30%, decreasing weight gain and linear growth of preterm infants over time (1, 5, 8).

Developing an alternative method to ensure donor milk's microbiological safety while preserving BSSL could lead to improved fat absorption and growth for premature infants. High-pressure processing (HPP), or high-hydrostatic pressure processing, applies pressure without heat, which inactivates bacteria and viruses in food by inhibiting their enzyme activity, damaging cell membranes, and inhibiting genetic transcription and translation (9). HPP treatment of human milk was able to maintain the bioactivity of BSSL, lysozyme and lactoferrin and minimize changes in the lipid profile compared with Holder pasteurization (6, 7). Gamma-cell irradiation (IR) can inactivate bacterial and viral pathogens by damaging their DNA (among other mechanisms) (10). IR treatment effectively sterilized human milk, however, it partially denatured milk protein, lactoferrin and IgA (11). Ultraviolet-C irradiation (UV-C) non-thermally inactivates bacteria and viruses by causing DNA structural damage with highest germicidal effect between 250 and 270 nm (12, 13) and can pasteurize donor milk (14). Preserved BSSL and alkaline phosphatase activities in human milk were observed after UV-C treatment compared with Holder pasteurization (15). The microbiological safety of human milk was ensured by HPP (6), IR (11), UV-C (15), and LTLT (16) in previous studies.

The amount of UV-C dosage required for UV-C pasteurization of human milk and the impact of UV-C, HPP, and IR pasteurization conditions on human milk BSSL activity needs further examination. Bacterial reduction in human milk by UV-C treatment was previously demonstrated for selected vegetative bacteria (15); however, the efficacy of UV-C to inactivate neonatal enteric pathogens and bacterial spores commonly isolated from pasteurized human milk has not been examined. Moreover, the resulting effect of UV-C on BSSL activity and the effect of IR on BSSL activity has not been determined. The objective of this study was to investigate the effect of UV-C exposure UV-C dosage on bacterial reduction including vegetative cells and spores in donor breast milk for microbiological safety and to determine the impact of alternative processing technologies on BSSL activity in donor breast milk.

## Methods

### Donor Milk Pooling

Frozen, untreated, and deidentified donor breast milk was donated by Northwest Mothers Milk Bank (Portland, OR, USA) to the Dallas laboratory in 2017. The informed consent procedure was managed by the Northwest Mothers Milk Bank and milk samples were collected from four donors. The untreated donor breast milk was stored at −20°C before pooling. Individual bags of frozen donor breast milk were placed at room temperature for 2 h then in a 4°C refrigerator for 48 h to thaw. After thawing, individual packs of the donor breast milk were pooled and divided into five aliquots for four treatments (LTLT, HPP, IR, and UV-C) and one control unprocessed sample (denoted as “untreated” in the **Results** section). All samples aliquots were placed back into a −20°C freezer for storage until treatments were carried out.

### Bacteriology

Bacterial species pertinent to transmission through donor breast milk were selected to identify UV-C processing conditions that would achieve a microbial reduction equivalent to that of thermal pasteurization. Bacteria used in this study included *Cronobacter sakazakii* (ATCC BAA-894), *Enterococcus faecium* (ATCC 8459), *Staphylococcus aureus* (138-CPS and 146-CPS), and spores of *Bacillus subtilis* (NRRL B-354, 356), *Paenibacillus macerans* (NRRL B-14029) and *Paenibacillus polymyxa* (NRRL B-510). A cocktail of five Listeria monocytogenes (ScottA, OSY-428, Ohio, California, ATCC 19115) strains were also evaluated for UV-C efficacy. *Bacillus subtilis, P. macerans*, and *P. polymyxa* were sporulated by incubation (each individually) in tryptic soy broth (Neogen, Lansing, Michigan) at 37°C for 4 days, and sporulation was confirmed by microscopy. All other bacteria were grown individually in tryptic soy broth with yeast extract (Neogen) at 37°C for 24 h. All 3 species of spore formers were combined into a single cocktail. Spores from all 3 species were combined together before mixing into the master cocktail with *C. sakazaki, E. faecium, S. aureus, L. monocytogenes* to achieve a final cell density of ~8-Log CFU/mL. One milliliter of the final cocktail was inoculated into a single sample of the 120-mL thawed donor breast milk. The mixture was stabilized at 4°C for 2 h prior to the UV-C treatment.

The inoculated milk was placed in a sterile 200-mL beaker wrapped with aluminum foil and stirred at 200 rpm throughout the UV-C treatment. A 9 W 254-nm UV-C twin tube lamp (MHFUV-H9WG23, Rexim, LLC, Watertown, MA) was submerged into the milk, touching the bottom of the beaker at a slight angle. Duplicate 1-mL aliquots of the milk were collected at 0 J/L (before UV-C exposure), and at UV-C dosage points up to 33,000 J/L after UV-C exposure. The collected aliquots for each time point were enumerated by standard serial dilution (Butterfield's Phosphate Buffer), spread plating on selective agar media and incubation at 37°C. Incubation times and selective media used were as follows: *C. sakazaki*, 24 h, MacConkey Agar (Neogen); *E. faecium*, 48 h, m-*Enterococcus* Agar (Neogen); *S. aureus*, 36 h, Mannitol Salt Agar (Neogen), *L. monocytogenes*, 48 h, Modified Oxford Listeria Agar Base (Neogen); and sporeformer, 36 h, Nutrient Agar (Neogen). Typical colonies were counted on each selective medium and converted to a Log_10_ scale.

### Donor Milk Processing

#### Thermal Processing—Holder (LTLT) Pasteurization

Three frozen 60-mL donor milk aliquots were thawed and placed in a 63°C water bath. When the milk temperature reached 63°C, it was held at 63°C water bath for 30 min to meet the non-profit milk bank definition of Holder pasteurization (17) and then transferred to an ice bath to quickly cool the milk 4°C. Heat-treated samples were frozen at −20°C for later analysis.

#### High-Pressure Processing (HPP)

Three frozen 60-mL donor milk aliquots were partially thawed, transferred to individual plastic bags and vacuum-sealed. Milk samples were shipped on dry ice to the HPP Validation Center at Cornell University (Geneva, NY) for high-pressure processing. Milk samples were treated at 550 MPa with a holding time of 5 min. Following HPP treatment, samples were packed immediately in dry ice and shipped back to Oregon State University the same day. Upon arrival at OSU, samples were frozen at −20°C for later analysis.

#### Irradiation (IR)

Three frozen 60-mL donor milk aliquots in glass vials were exposed to 2.5 Mrads of gamma cell irradiation from a cobalt-60 source at the Oregon State University Radiation Center. The sample chamber temperature was 32.2°C for the duration of 5-h treatment, thus the samples were slowly thawed during the treatment. The samples were stored at −20°C for further analysis.

#### Ultraviolet-C (UV-C)

Three frozen 60-mL donor milk aliquots were thawed and transferred into a 150-mL beaker wrapped with aluminum foil with a submerged 254 nm UV-C light with magnetic stirring at 400 rpm. Triplicate 1-mL aliquots of the sample were collected from 0 to 33,000 J/L of UV-C exposure and immediately stored at −20°C. The output power of UV-C lamp was 1.1 W measured at the middle of the lamp by a UV-C light meter (Avantes AvaSpec-ULS204BCL-EVO multiplex detector) after a cosine corrector on the end of the fiber optic probe. UV-C dosage (J/L) was calculated by multiplying UV-C output power (W) and treatment time (s) divided by the total sample volume (L).

#### Bile Salt-Stimulated Lipase (BSSL) Activity

To determine BSSL activity, the concentration of *p*-nitrophenol cleaved from *p*-nitrophenyl myristate by BSSL was measured spectrophotometrically by a modified published method (18). The untreated and treated donor breast milks were thawed on ice for 30 min and 10 μL of each were diluted 1:100 in water. A buffer solution of 0.4 M Tris-HCl, 116 mM sodium cholate in water was prepared with mixing at 37°C and adjusting to pH 8.0 with 6 M NaOH. A solution of 14 mM *p*-nitrophenyl myristate in aqueous 0.5 M 2-methoxyethanol was added to the buffer solution. An aliquot of 33.3 μL of each diluted sample, 83.3 μL of water and 83.3 μL of buffer solution were mixed in a 96-well plate. Production of *p*-nitrophenol was measured by a spectrophotometer monitoring absorbance at 405 nm from 30 s up to 15 min of incubation at a constant temperature of 37°C. A *p*-nitrophenol standard curve was constructed via serial dilution with a range from 12 to 1,500 μM. To calculate the BSSL activity (U/mL), the concentration of produced *p*-nitrophenol was divided by an incubation time of 30 s where the highest level of substrate existed.

### Statistical Analysis

One-way ANOVA with Tukey's multiple comparisons-test was used to compare BSSL activity among treatment groups and the untreated milk control with a significance level of 0.05. Statistical analysis was conducted with Prism 8.

## Results

### UV-C Treatment: Bacterial Reduction and BSSL Activity

**Figure 1:**
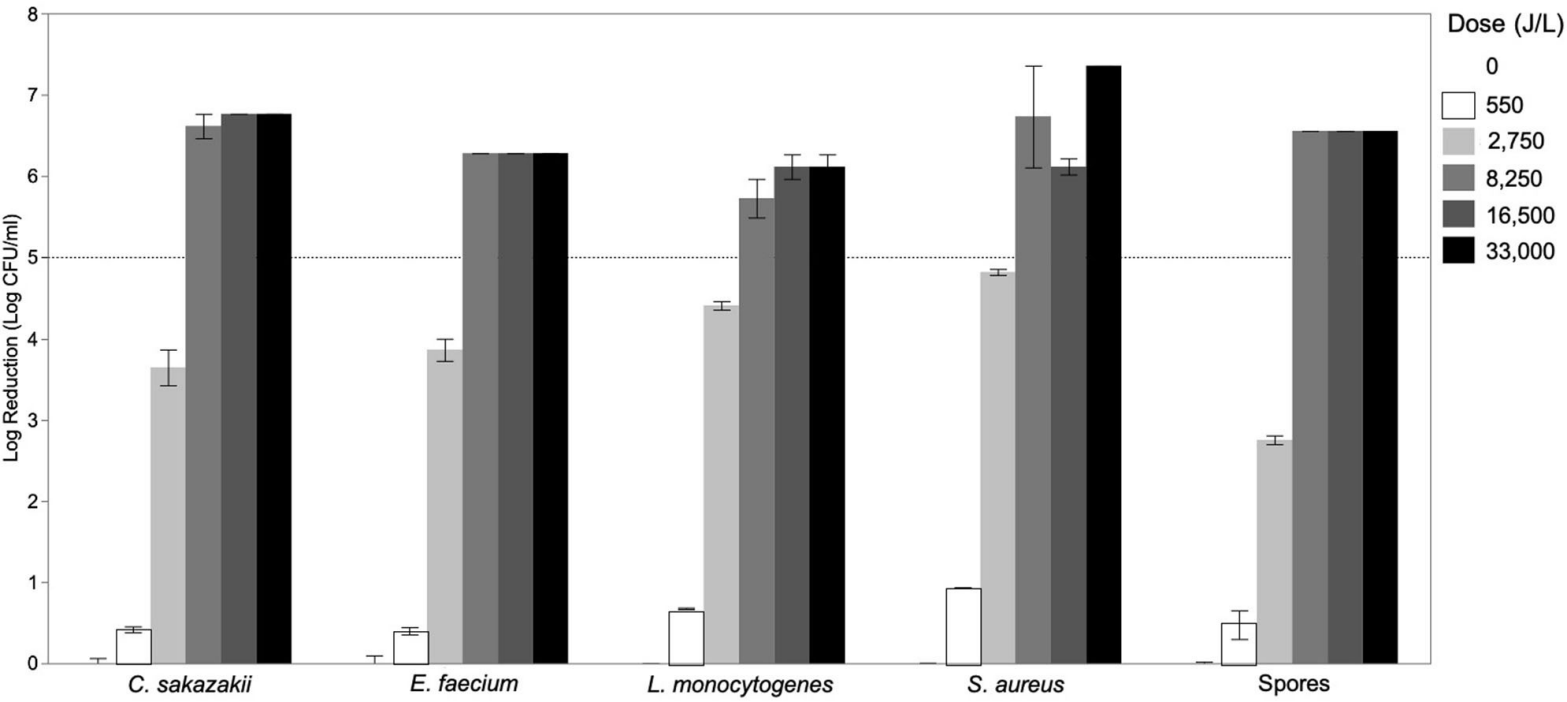
Inactivation of *Cronobacter sakazakii, Enterococcus faecium, Listeria monocytogenes, Staphylococcus aureus*, and spores of *Bacillus subtilis, Paenibacillus macerans*, and *Paenibacillus polymyxa* in donor breast milk as a function of UV-C exposure dose (0–33,000 J/L). Data is presented as the log reduction of each UV-C-time combination with the error bars indicating the standard error of the mean of technical replicates (*n* = 2).

The effect of UV-C treatment of donor breast milk with different UV-C exposure dosages (0–33,000 J/L) on reduction of various vegetative bacteria and spores was determined (Figure 1). *Cronobacter sakazakii* was known to contaminate expressed mother's milk and cause infant sepsis, and can contaminate formula (19). *Enterococcus* was present in 16% of unpasteurized donor milk samples (16) and *E. faecium* specifically was found in human milk (20). Neonatal pathogen *S. aureus* also was found in untreated donor milk (16). A case report indicates that *L. monocytogenes* present in mother's milk caused neonatal sepsis (21). *Bacillus* sp. were the most common contaminant after pasteurization of human donor milk (16). Though *Paenibacillus* has not been identified specifically in human milk, the genus is commonly associated with food spoilage and is present in raw and heat-treated bovine milk (22, 23). Minimal reductions (<1.0-log reduction) were achieved with a UV-C exposure dosage of 550 J/L. After 2,750 J/L of UV-C exposure, all bacterial targets had been significantly reduced, but the degree of inactivation differed by species. As expected, spores were more resistant to UV-C treatment (2.75-log reduction) as compared to vegetative bacteria (3.64–4.82-log reduction). All bacterial targets (vegetative and spores) were inactivated by >5-log CFU/mL at the 8,250 J/L UV-C dosage. Increasing the UV-C exposure dosage to 16,500–33,000 J/L may increase the inactivation; however, this was not demonstrated in this study as any survivors at 33,000 J/L were below the detection limit of our methodology (1 log CFU/mL).

**Figure 2:**
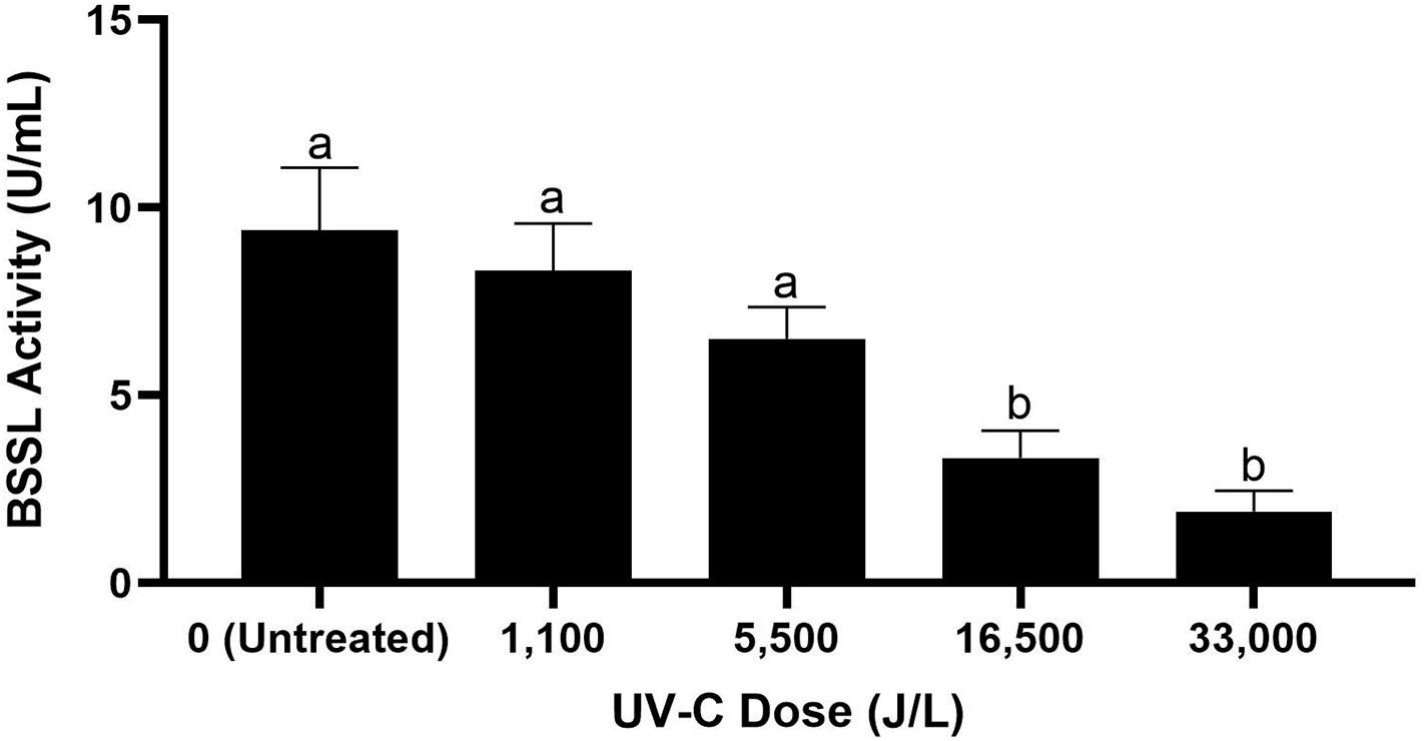
Bile salt-stimulated lipase activity in untreated donor breast milk and donor milk treated by UV-C at different exposure doses from 0 to 33,000 J/L. Different letters indicate that the samples are significantly different at *p* < 0.05.

The BSSL activity of untreated donor breast milk was 9.4 U/mL. Donor breast milk treated by UV-C had BSSL activity of 8.3 U/mL at 1,100 J/L of UV-C treatment, 6.5 U/mL at 5,500 J/L, 3.3 U/mL at 16,500 J/L, and 1.9 U/mL at 33,000 J/L (Figure 2). Donor milk BSSL activity was significantly reduced after 16,500 J/L of UV-C exposure; however, further UV-C treatment (up to 33,000 J/L) did not result in further significant reduction in BSSL activity (*p* < 0.05).

### Comparing Retention of BSSL Activity Across Alternative Pasteurization Methods

The FDA stipulates that pasteurization must meet a 5-log reduction (99.999% destruction) of the pathogenic microorganism most commonly found to contaminate a product (24). Donor human milk is not regulated by the FDA, however, to meet the equivalent of Holder pasteurization which is currently used by Human Milk Banking Association of North America (HMBANA) with a significant margin of safety, the 16,500 J/L of UV-C treatment was selected to compare with Holder pasteurization, IR, HPP, and LTLT for retention of BSSL activity in donor breast milk.

**Figure 3:**
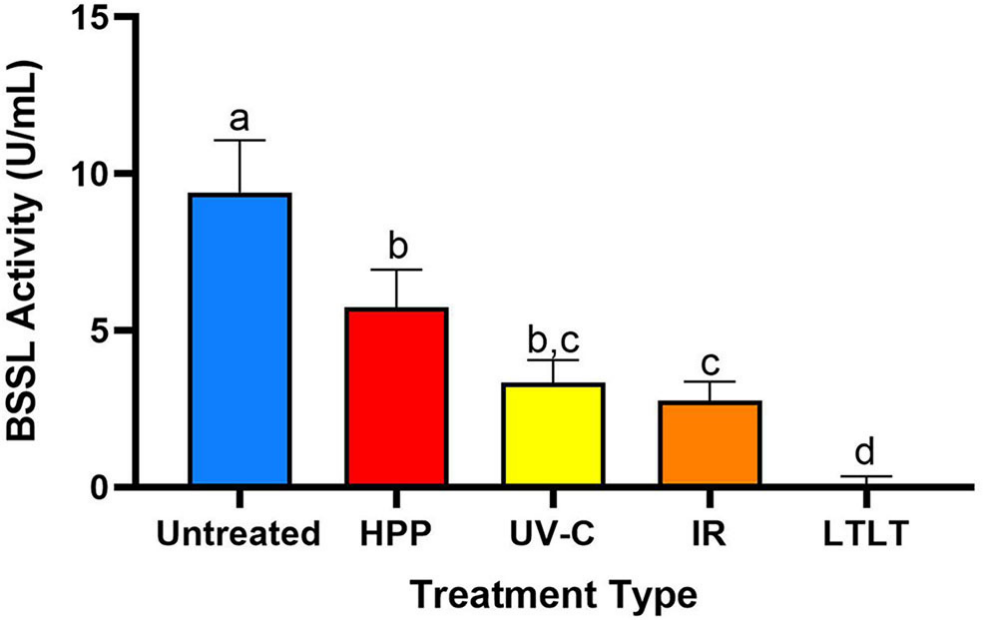
Bile salt-stimulated lipase activity in untreated donor breast milk and donor milk treated by HPP, UV-C (16,500 J/L), IR and LTLT. Different letters indicate that the samples are significantly different at *p* < 0.05.

The untreated donor breast had the highest BSSL activity (9.4 U/mL), followed by HPP-, UV-C-, IR-, and LTLT-treated donor breast milk, 5.8, 3.3, 2.7, and 0.0 U/mL, respectively (Figure 3). Compared with untreated donor breast milk, BSSL activity decreased 38.4% in HPP-, 64.7% in UV-C, 70.8% in IR-, and 99.7% in LTLT-treated donor breast milk (*p* < 0.05). These findings indicate that though human milk BSSL activity decreased during all tested pasteurization techniques, HPP treatment preserved the highest degree of activity and LTLT preserved the least.

## Discussion

Fat digestion is essential for neonatal infants and breast milk BSSL plays an important role in the digestion of lipids. As mothers of neonates often cannot produce adequate amounts of breast milk, these infants are fed either entirely or supplementally donor breast milk processed by milk banks. Holder pasteurization (LTLT) is the current process to ensure the microbiological safety of donor milk at all non-profit milk banks. We found that Holder pasteurization reduced BSSL activity 99.7% compared with untreated donor breast milk (Figure 3), which was in agreement with a previous study demonstrating that only 1% BSSL activity remained in human donor milk after Holder pasteurization (14). Current processing methods at milk banks destroy the beneficial effect of BSSL that would support neonatal fat digestion, a key nutrient for growth. Indeed, infants fed pasteurized mother's milk had lower fat absorption and lower knee-heel length (5) and weight gain (8) compared with infants fed untreated mother's milk.

Gamma cell irradiation (IR) was tested as a potential alternative to LTLT. During IR exposure, bacteria are destroyed by degradation of their DNA, which inhibits replication. This method has been adopted to ensure food safety for a variety of products by over 40 countries and endorsed as safe by the American Medical Association, World Health Organization, and Food and Drug Administration (25). IR can completely sterilize milk samples (11). As the BSSL activity in IR-treated donor breast milk was higher than that in LTLT-treated donor breast milk, IR could be a candidate for an alternative treatment of LTLT. Though less destructive than LTLT, IR processing decreased BSSL activity 70.8% compared with activity in untreated donor breast milk. IR treatment may not be feasible for milk banks because of the long processing time and significant capital expense.

UV-C exposure inactivates microorganisms by damaging their DNA (12). We demonstrated that UV-C irradiation at >8,250 J/L effectively reduced >5-log CFU/mL of a cocktail of pathogens and relevant bacterial spores in human milk. We also demonstrated that BSSL activity is significantly reduced at UV-C dosage between 5,500 and 16,500 J/L (Supplementary Table). UV-C treatment at 16,500 J/L decreased BSSL activity by 64.7%; however, this process retained significant BSSL activity which is an improvement from the current LTLT process. Two previous publications reported no loss of BSSL activity after UV-C treatment (15, 26). Christen et al. (15) reported retention of BSSL activity (1% reduction) with low UV-C dosage (2,084–4,863 J/L) which aligns with the current results of no significant BSSL reduction at ≤ 5,500 J/L (15). However, a recent publication reported significant degradation of BSSL activity with the 25 min required to achieve the desired total bacterial load reduction which were incubated in MacConkey agar and blood agar (14). This variation in outcome is likely due to variations in UV-C exposure dosage amongst the treatments as well as exposure and matrix differences. UV-C processing could be implemented commercially to provide donor milk with at least somewhat improved retention of BSSL activity. Bovine milk has successfully been pasteurized with a commercial continuous flow, in-line UV-C system (27). As varying fat contents of the bovine milk affected the log reduction of pathogens, further studies would be needed to determine standard parameters for human milk using this system. UV-C treatment systems are fairly inexpensive (~$10,000–$15,000), have low energy consumption, and require little ongoing maintenance (28).

For HPP, microbiological safety of bovine milk has been demonstrated at pressures between 400 and 800 MPa for 5–10 min (9). HPP at 400 MPa for 4 and 2 min provided 8-log reduction of *Streptococcus agalactiae* and *Listeria monocytogenes*, respectively, in human milk (29). HPP at 500 MPa for 8 min has also been demonstrated to increase the number of culture negative (<1 × 10^3^ CFU/L) donor human milk samples without destruction of BSSL activity (14). The HPP conditions used in our study (550 MPa, 5 min) best preserved BSSL activity compared with IR, UV-C, and LTLT. Moreover, HPP has been previously shown to improve the retention of other bioactive milk proteins such as lactoferrin, lysozyme and immunological components compared with LTLT (6, 9, 14). Additionally, HPP minimized changes in the free fatty acid content and oxidative stability of human milk compared to Holder pasteurization (7). The structure of casein micelles and alpha-lactalbumin, however, is altered after HPP (30), which could alter the function, digestion and release of bioactive peptides in the infant. Though BSSL and some bioactive milk proteins are better preserved by HPP, the entire array of bioactive milk proteins must be investigated for structural and functional retention after HPP, as each could be differentially affected by HPP, a process that affects non-covalent bonds (i.e., ionic, hydrophobic, and hydrogen bonds) and thus can alter the secondary, tertiary, and quaternary structure of proteins (31). Proteomics and multiplex ELISA among other techniques could help answer these questions.

Implementation of HPP treatment by milk banks could be complicated by the cost of the HPP equipment. Purchase and installation of a 55-L HPP system, which would be suitable for milk bank processing volumes, can cost over $700,000 (32). Milk banks could, however, ship samples to external HPP facilities for processing. Though HPP may have higher implementation costs than LTLT, the significant retention of BSSL activity and potential to improve the fat digestion and growth of preterm infants makes this approach worth further exploration.

As an alternative strategy to preserving BSSL in donor milk, previous studies have examined supplemental recombinant BSSL for preterm infants. However, the clinical study showed no differences in growth rate between recombinant BSSL-fed and placebo groups, except for small for gestational age pre-term infants (33). A possible explanation for this finding is that the supplemented recombinant BSSL might not be as stable in the infant intestine as naturally occurring BSSL. For example, another endogenous milk protein, IgG, survived intact across *ex vivo* infant digestion to much greater extent than a recombinant human IgG (34). Based on this result and an observed increase in adverse events in the supplemented group, developing an alternative approach to ensuring donor milk safety while preserving BSSL activity may be a preferable strategy for improving premature infant growth.

The current research demonstrates the potential for UV-C, IR, and HPP to retain BSSL activity in donor human milk using processes that achieve pasteurization. If milk banks implemented one of these alternative processing techniques, donor human milk could improve lipid absorption and better promote growth in preterm infants. Though HPP preserves BSSL better than other processing methods, further research is needed to examine its effects on other milk components and on infant health outcomes. Future studies should investigate the effect of feeding HPP-treated donor breast milk on lipid absorption, growth, and health status in preterm infants.

## Data Availability Statement

The raw data supporting the conclusions of this article will be made available by the authors, without undue reservation.

## Author Contributions

JK guided and edited the manuscript. AV conducted IR-, LTLT-, and HPP-treatments. AV and YQ developed the BSSL protocol. MH and JY conducted the UV-C treatments and BSSL experiments. BS conducted the bacteriology study. DD and JW-C designed the concept, acquired funding, provided guidance for the study, and reviewed the final manuscript. All authors contributed to the article and approved the submitted version.

## Conflict of Interest

The authors declare that the research was conducted in the absence of any commercial or financial relationships that could be construed as a potential conflict of interest.
