# Supplementary material for: Bile Salt-Stimulated Lipase Activity in Donor Breast Milk Influenced by Pasteurization Techniques
Source: Front Nutr. 2020 Nov 12;7:552362. doi: 10.3389/fnut.2020.552362 (PMC7689290; doi:10.3389/fnut.2020.552362)
Supplement: Supplementary file 1 [file Table_1.DOCX]

|  | Christen et al. (2013) | Koh | Koh |
| --- | --- | --- | --- |
| Initial BSSL activity (U/mL) | 116.5 ± 36.6 | 9.4 ± 1.7 | 9.4 ± 1.7 |
| BSSL activity after UV-C (U/mL) | 115.3 ± 34.4 | 6.5 ± 0.8 | 3.3 ± 0.7 |
| BSSL activity reduction (%) | 1 | 30.5 | 64.7 |
| UV-C dosage (J/L) | 4,863 | 5,500 | 16,500 |

Supplementary table. A comparison of initial BSSL activity, BSSL activity after UV-C treatment, BSSL reduction and UV-D dosage from previous study.

Mean ± standard deviation. Bile salt-stimulated lipase (BSSL)

Reference

Christen, L., Lai, C.T., Hartmann, B., Hartmann, P.E., and Geddes, D.T. (2013). Ultraviolet-c irradiation: a novel pasteurization method for donor human milk. *PLoS One* 8**,** e68120.
